# Assessment of Sensory Processing Issues in Children with Neurodevelopmental Disorders and Experiences of Maltreatment

**DOI:** 10.3390/children11020216

**Published:** 2024-02-07

**Authors:** Ayako Ide-Okochi, Mu He, Tomofumi Tokieda, Satsuki Nakamura, Nobutomo Matsunaga

**Affiliations:** 1 Faculty of Health Sciences, Kumamoto University, Kumamoto 862-0976, Japan; nakamura_s@kumamoto-u.ac.jp; 2 Graduate School of Health Sciences Education, Kumamoto University, Kumamoto 862-0976, Japan; 3 Graduate School of Science and Technology, Kumamoto University, Kumamoto 860-8555, Japan; 4 Faculty of Advanced Science and Technology, Kumamoto University, Kumamoto 860-8555, Japan

**Keywords:** gaze, interoception, autism spectrum disorder, attention deficit/hyperactivity disorder, maltreatment, trauma, virtual reality, sensory processing, children

## Abstract

This study aims to identify the sensory characteristics of children with both developmental disabilities such as ASD and ADHD and a history of child maltreatment (DM group), children with developmental disabilities (DD group), and typically developed children (TD group). The sensory characteristics of 24 children were assessed through virtual reality and self-administered questionnaires. The results elucidated that the duration of gazing at the “teacher” (60–75 s) was significantly different with the DM group’s gaze being shorter than the DD group’s gaze (*p* = 0.042). The duration of the “others” gaze (45–60 s) was also significantly different with the DM group’s gaze being longer than the DD and TD groups’ gaze (*p* = 0.018; *p* = 0.030). Additionally, the scores for tactile sensitivity, taste/smell sensitivity, under-responsive/seeks sensation, and the total short-term sensory profile were significantly different between the DM-DD and DD-TD groups. The scores of the heart rate perception test and Multidimensional Assessment of Interoceptive Awareness were significantly lower in the DM and DD groups. In conclusion, children who have experienced trauma and developmental disabilities may have different sensory characteristics than children with only developmental disabilities or none, implying the need for further research and tailored care.

## 1. Introduction

Attention deficit/hyperactivity disorder (ADHD) and autism spectrum disorder (ASD) are the prevalent neurodevelopmental disorders. According to a recent cohort study in Sweden, the prevalence of ADHD and ASD was 7.6% and 1.1%, respectively [1]. In Japan, the percentage of children suspected of having ASD, ADHD, or learning disabilities (LD) among children aged 6–15 years was 8.8% in 2022 [2], a 2.3% increase compared to 6.5% in the 2012 national survey [3]. In the 2022 national survey, 4.7% of pupils were reported to possibly have ASD and/or ADHD and were assessed as demonstrating significant behavioural difficulties [2]. According to a review, studies have estimated that between 37% and 85% of children with autism exhibit symptoms of attention deficit hyperactivity disorder (ADHD) [4]. Although inattention in ASD may have different neurological features than in ADHD, a dual diagnosis is very common [5]. This study addresses children with ASD and/or ADHD when referring to children with developmental disabilities.

Developmental disabilities are a risk factor for maltreatment; according to a WHO fact sheet, having special needs and neurodevelopmental disorders were children’s risk factors [6]. Maltreatment is one of the adverse childhood experiences that has a significant impact on child development and has remained steadily high in recent years. The WHO global status report revealed that nearly 3 in 4 children or 300 million children aged 2–4 years regularly suffer physical punishment and/or psychological violence at the hands of parents and caregivers [7]. For example, 205,044 cases of child maltreatment were reported in Japan in 2020, up from 122,575 cases in 2016 [8]. In the United States (U.S.), the number of victims of maltreatment was 588,229 in 2021 [9]. Not only do abuse and maltreatment affect children’s deaths in the U.S., reportedly every 2.46 of 100,000 children in 2021, but they may also go unrecognised [9].

Globally, a significant proportion of children who experience child abuse and maltreatment are considered to have neurodevelopmental disorders. Sullivan et al. [10] found that the incidence of maltreatment in children with disabilities was 31%, compared to 9% in nondisabled children, 3.4 times higher than that in children without disabilities. Historically, people with developmental disabilities have been particularly vulnerable to abuse by parents and institutional staff [11]. Furthermore, ASD and ADHD were significantly associated with deaths due to neglect in 2010–2019 statewide [12]. Therefore, early support for children with developmental disabilities is essential to prevent trauma from abuse.

Children and adolescents with developmental disabilities or experiences of abuse often have sensory processing disorders. Parent surveys indicate that 45–95% of patients with ASD exhibit a high frequency of nonstandard sensory behaviours [13]. A meta-analysis indicated significantly higher differences in the presence and frequency of sensory symptoms between the ASD and typical groups, with the largest differences being in “under reactivity”, followed by “over reactivity” and “sensory seeking” [13]. Moreover, sensory processing problems in children with ADHD are more common than in children with typical development [14]. Additionally, a correlation between abused children and sensory processing disorders has been noted: a review of 13 articles indicated that child trauma victims exhibit “sensory modulation dysfunction” [15]. Atchison [16] reported that more than half of the children exhibited symptoms of sensory modulation disorders based on data from a Sensory Profile (SP) questionnaire administered to 900 children referred to a trauma assessment centre.

Sensory processing deficits may affect higher-order social problems and academic performance; children with ADHD and/or ASD have difficulty recognising speech through hearing in the presence of noise and struggle in school [17]. In children with ASD, visual search patterns in social situations differ from the standard patterns, and a higher intensity of sensory problems is associated with more pronounced social difficulties and poorer adaptive functioning [18]. Furthermore, childhood maltreatment has significant effects on brain development, with certain types of maltreatment producing changes in brain regions (auditory, visual, and somatosensory cortices) and pathways that process and transmit aversion experiences [19]. Academic difficulties are apparent in residents of children’s homes, where approximately half of the children admitted to the facility have experienced abuse [20]. Furthermore, inferior sensory perception has been associated with anxiety in individuals with developmental disabilities [21]. Survivors of childhood abuse had a relatively high prevalence of anxiety and other psychopathologies [19].

Therefore, sensory idiosyncrasies due to neurodevelopmental disorders and abusive experiences affect children’s social and academic skills, which in turn affect their subsequent well-being and therefore need to be understood at an early stage. We developed a system that enables visual search in a simulated classroom environment reproduced by virtual reality (VR) [22]. Children with ASD and/or ADHD stared at the teacher significantly more than typically developing children but tended to have poorer listening comprehension. Moreover, children with developmental disabilities were significantly less somatosensory than typically developing children in six of the eight subdomains of the Multidimensional Assessment of Interoceptive Awareness (MAIA). However, in a previous study, preschoolers with ASD spent less time staring at the object the teacher was pointing to when looking at a classroom picture than typically developing preschoolers [23], and children with ASD and ADHD had different sensory systems, such as oral and visual processing scores, compared to typically developing children [24]. Additionally, a large sample study found that 53% of child victims of trauma assessed using the SP showed differences in sensory modulation, with 63% of participants being unresponsive or sensation-seeking, while those who were tactile (42% of the sample) and experienced auditory filtering (66% of the sample) were hyperresponsive [16]. Thus, it can be inferred that children with developmental disabilities and trauma from abuse may have some sensory and response differences but in different domains. Some senses may particularly differ between abused children and those with developmental disabilities. We hypothesised that children with developmental disabilities and a history of maltreatment would have different sensory characteristics from children with developmental disabilities only or typically developing children. The purpose of this study is to identify the sensory characteristics of three types of children, children with developmental disabilities, children with developmental disabilities and a history of abuse, and standard children, and to provide suggestions for the prevention of school maladjustment.

Our previous preliminary research paper reported a system that detects pupils’ gaze patterns in a classroom environment reproduced by virtual reality (VR) [22]. Although the developmentally disabled and typically developing groups of children are historically contrasting samples [23,25], this paper focuses on children who have experienced maltreatment as well.

## 2. Materials and Methods

### 2.1. Participants

The participants were ten children with developmental disabilities and a history of parental abuse (DM group), seven children diagnosed with developmental disabilities (DD group), and seven children in the control group with typical development (TD group). The diagnoses of the DM and DD groups were ASD and/or ADHD. All children in the DM group were recruited by psychologists in 2022 after the research purpose and ethical considerations were explained to them. All the recruited children resided in a child psychological treatment facility, as defined in Article 43-2 of Child Welfare Act [26]. This facility admits children who have difficulties in adapting to social life, their family environment, friendship in schools, or other environmental reasons. Several psychologists of the studied facility diagnosed the DM group participants based on the DSM-IV criteria and ICD-10, under consultation with the child guidance centre, which staffs physicians, psychologists, and public health nurses. In Japan, the DSM-IV or ICD-10 are used for judicial purposes [27], and these diagnostic criteria were used in this study because a placement in a child psychological treatment institution is equivalent to an administrative measure [26]. The DM group included six children with ASD and ADHD and three with ADHD only. For the DM group, the head of the facility explained the study to the participants and obtained written consent. In Japan, when an abused child is placed in a children’s home, the head of the home is allowed to give surrogate consent. All children in the DD group were recruited by physicians from the Department of Paediatrics, University Hospital of Japan, in 2020–2021. Two physicians diagnosed the participants based on the DSM-V criteria [28]. The DD group included six children with ASD and ADHD, one child with ASD only, and one child with ADHD only. Finally, among participants in the DM and DD group, 12 children with ASD and ADHD, 1 child with ASD, and 4 children with ADHD were included. Participants in the control group were neither diagnosed nor suspected of having developmental disabilities; the TD group was also given a written explanation, and consent was obtained in writing. All groups were informed by the researcher on the day of data collection about the purpose, content, and burden of the study, as well as the protection of personal information and assurance of withdrawal, and consent was obtained.

All participants had to have no vision problems. Participants with epilepsy, intellectual disability, and neurological disorders affecting movement were excluded from the study.

The intelligence quotient (IQ) of the participants in the DM and DD groups was verified as 70 or higher by the facility director (a licenced psychologist) and the physician, respectively. The parents also confirmed that none of the children in the TD group had intellectual disabilities. The same performance test (quiz) was administered to all the participants.

### 2.2. Data Collection

#### 2.2.1. Gathering of Eye Gaze Data Using a VR Classroom

A VR classroom was designed [22]. We measured eye gaze during visual and auditory interference stimuli using the VIVE Pro Eye. Individual gaze time data were summed for each group, and the mean value for each group was calculated.

#### 2.2.2. Short Sensory Profile

The SP was developed in the United States (U.S.) by Winnie Dunn. The SP is a parent-reported measure of a child’s response to sensory experiences that occur in everyday life [29]. The instrument was designed to capture sensory-processing behaviours that indicate an overreaction (low threshold) or under reaction (high threshold) to sensory experiences. The instrument consists of 125 items and was standardised on a sample of 1037 U.S. children aged 3 to 10 years. Ermer and Dunn [25] demonstrated that this SP could effectively discriminate children with ASD (*n* = 38) or ADHD (*n* = 61) from those without disabilities (*n* = 1075). The Japanese version of the Short Sensory Profile has been tested for reliability and validity [30]. Parents and caregivers reported the frequency of sensory problems using a 38-item Short Sensory Profile (SSP) consisting of seven subscales (tactile hypersensitivity, taste and smell hypersensitivity, motor hypersensitivity, lack of response and sensory seeking, auditory filtering, low energy and weakness, and visual and auditory hypersensitivity). Hypersensitivity or lack of response was assessed; parents responded to the SP for groups DD and TD and the children’s centre staff for group DM. Each subscale and the total score were calculated.

#### 2.2.3. Interoception

The participants’ interoception was measured using both Schandrys’ [31] heartbeat tracking task and the self-administered questionnaire of MAIA [32]. The Japanese version of MAIA has been validated previously [33]. The MAIA is a 32-item multidimensional instrument with eight subscales: awareness, distraction-free, worry-free, attention control, emotional awareness, self-control, body listening, and trust. The MAIA is a 5-point Likert scale, where the mean is derived from the total score with respect to each subscale.

#### 2.2.4. Performance Test (Quiz)

A quiz was created and used to assess the comprehension of the “teacher’s explanations in the VR classroom” [22]. The self-administered quiz consisted of five questions from the teacher’s explanation in a 90 s VR video, which the children answered on their own.

### 2.3. Data Analysis

Descriptive statistics were performed on the attributes of the three groups (age, sex), the gazing time per 15 s, SSP scores, quiz scores, the heartbeat perception test and MAIA; after confirming the normality of the data using the Shapiro–Wilk test, three-group comparisons were made using the Kruskal–Wallis test, and the Bonferroni correction was used to compare the two groups. A correlation of 0.05 was treated as statistically significant. All statistical analyses were performed using the IBM SPSS statistical software Version 28.

## 3. Results

There were eight patients in the DD group (six males), nine in the DM group (three males), and seven in the TD group (five males), with an overall mean age of 13.08 ± 1.84 years (Table 1). There were no significant differences in age or sex among the three groups.

### 3.1. Group Differences in Gaze (Table 2, Figure 1)

In the DM group, the time to look at the “teacher” decreased from about 8.8 s to about 3.8 s when moving from 30–45 s to 45–60 s, and remained at about 4.9 s when moving from 60–75 s. The TD group consistently had the longest gaze time at the bulletin board from 0–60 s.

There was a significant difference between the three groups in the duration of gazing at the “teacher” between 60 and 75 s (*p* = 0.028), with the DM group gazing for a significantly shorter time than the DD group (*p* = 0.042). There was a significant difference between the three groups in the time spent looking at the “other” between 45 and 60 s (*p* = 0.007), with the DM group looking at it significantly longer than the DD and TD groups (*p* = 0.018; *p* = 0.030).

**Table 2:** Comparison of three groups’ gazing time (*n* = 24).

	Total (*n* = 24)	DM (*n* = 9)	DD (*n* = 8)	TD (*n* = 7)
Characteristics	Mean ± SD	Mean ± SD	Mean ± SD	Mean ± SD
Gazing time from 0 to 15 s (second)				
Teacher	9.25 ± 3.50	8.01 ± 3.96	10.83 ± 3.33	9.05 ± 2.72
	*p* = 0.486			
Notice	1.17 ± 1.14	1.03 ± 1.36	0.98 ± 1.13	1.57 ± 0.89
	*p* = 0.432			
Others	4.58 ± 3.28	5.96 ± 3.63	3.19 ± 2.46	4.38 ± 3.35
	*p* = 0.350			
Gazing time from 15 to 30 s (second)				
Teacher	8.51 ± 3.95	8.04 ± 4.30	10.03 ± 3.96	7.39 ± 3.45
	*p* = 0.340			
Notice	1.48 ± 1.57	1.56 ± 2.02	1.23 ± 1.31	1.68 ± 1.35
	*p* = 0.842			
Others	5.01 ± 3.83	5.41 ± 3.67	3.74 ± 3.86	5.94 ± 4.19
	*p* = 0.395			
Gazing time from 30 to 45 s (second)				
Teacher	9.57 ± 3.75	8.79 ± 4.77	11.63 ± 2.36	8.21 ± 2.87
	*p* = 0.132			
Notice	1.11 ± 1.09	1.11 ± 1.34	0.87 ± 1.13	1.37 ± 0.70
	*p* = 0.332			
Others	4.33 ± 3.58	5.09 ± 4.23	2.51 ± 2.37	5.42 ± 3.47
	*p* = 0.161			
Gazing time from 45 to 60 s (second)				
Teacher	6.44 ± 3.68	3.76 ± 1.86	8.19 ± 4.12	7.86 ± 3.21
	KW = 6.394			
	*p* = 0.041			
Notice	2.30 ± 1.84	1.28 ± 1.90	2.71 ± 1.43	3.15 ± 1.77
	*p* = 0.080			
Others	6.26 ± 4.38	9.96 ± 2.41	4.10 ± 4.43	3.99 ± 3.21
	KW = 9.810	D^DM-DD^ = −9.458		D^TD-DM^ = −9.190
	*p* = 0.007	*p*^DM-DD^ = 0.018		*p*^TD-DM^ = 0.030
Gazing time from 60 to 75 s (second)				
Teacher	7.26 ± 3.24	4.92 ± 3.00	8.90 ± 2.47	8.38 ± 2.80
	KW = 7.138	D^DM-DD^ = 8.444		
	*p* = 0.028	*p*^DM-DD^ = 0.042		
Notebook	0.75 ± 0.95	0.14 ± 0.2	1.11 ± 1.21	1.13 ± 0.89
	*p* = 0.052			
Others	6.32 ± 3.14	8.14 ± 3.52	4.99 ± 2.48	5.49 ± 2.44
	*p* = 0.149			
Quiz score	78.33 ± 22.00	75.56 ± 21.86	70.00 ± 23.9	91.43 ± 15.74
	*p* = 0.145			

DD: children with developmental disabilities. DM: foster home residents with developmental disabilities and experiences of maltreatment. TD: children with typical development. KW: Kruskal–Wallis test. D: Dunn–Bonferroni test. We have shown Bonferroni-corrected *p*-values.

**Figure 1:**
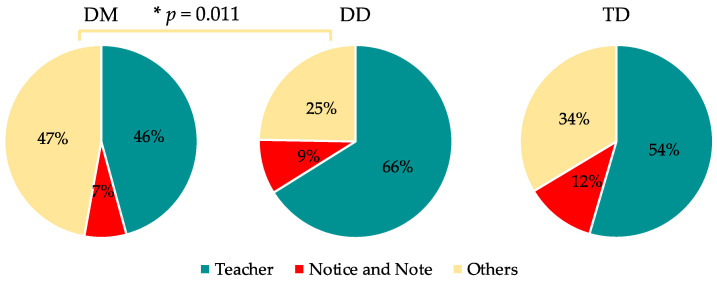
Percentage of time spent per gazed objects from 0 to 75 s (*n* = 24). Asterisk represent significant differences, * *p* < 0.05.

### 3.2. Difference in SSP (Table 3)

Comparisons between the three groups exhibited significant differences in tactile sensitivity (*p* = 0.032), taste/smell sensitivity (*p* = 0.013), under-responsive/seeks sensation (*p* = 0.009), and total SSP (*p* = 0.002). The Bonferroni correction results indicated that the DD group scored predominantly higher than the DM group in tactile sensitivity (*p* = 0.028). In the other subsections, the DD group scored significantly higher than the TD group (taste/smell sensitivity (*p* = 0.009), under-responsive/seeks sensation (*p* = 0.007), and auditory filtering (*p* = 0.006)). For all the SSP scores, the DD group scored predominantly higher than the DM and TD groups (*p* = 0.025; *p* = 0.003, respectively).

**Table 3:** Comparison of three groups’ SSP scale scores (*n* = 24).

	Total (*n* = 24)	DM (*n* = 9)	DD (*n* = 8)	TD (*n* = 7)
Characteristics	Mean ± SD	Mean ± SD	Mean ± SD	Mean ± SD
SSP				
Tactile Sensitivity	9.71 ± 2.27	8.33 ± 1.41	11.13 ± 1.81	9.86 ± 2.79
	KW = 6.912	D^DM-DD^ = 8.757		
	*p* = 0.032	*p*^DM-DD^ = 0.028		
Taste/Smell Sensitivity	6.42 ± 2.39	6.11 ± 1.62	8.38 ± 2.72	4.57 ± 0.79
	KW = 8.763		D^DD-TD^ = −10.643	
	*p* = 0.013		*p*^DD-TD^ = 0.009	
Movement Sensitivity	3.96 ± 1.46	3.33 ± 1.00	4.25 ± 1.75	4.43 ± 1.51
	KW = 4.494			
	*p* = 0.106			
Under-Responsive/Seeks Sensation	11.92 ± 5.06	11.67 ± 5.89	15.38 ± 3.89	8.29 ± 1.70
	KW = 9.492		D^DD-TD^ = −10.911	
	*p* = 0.009		*p*^DD-TD^ = 0.007	
Auditory Filtering	14.71 ± 6.17	14.33 ± 6.38	19.38 ± 3.58	9.86 ± 4.49
	KW = 9.474		D^DD-TD^ = −11.223	
	*p* = 0.009		*p*^DD-TD^ = 0.006	
Low Energy/Weak	12.96 ± 7.14	11.44 ± 7.45	17.38 ± 7.23	9.86 ± 4.45
	KW = 5.279			
	*p* = 0.071			
Visual/Auditory Sensitivity	7.13 ± 2.46	6.44 ± 2.01	8.75 ± 2.76	6.14 ± 1.86
	KW = 6.123			
	*p* = 0.047			
SSP Total	66.79 ± 19.67	61.67 ± 20.49	84.63 ± 8.96	53.00 ± 12.00
	KW = 12.325	D^DM-DD^ = 9.049	D^DD-TD^ = −12.152	
	*p* = 0.002	*p*^DM-DD^ = 0.025	*p*^DD-TD^ = 0.003	

DD: children with developmental disabilities. DM: foster home residents with developmental disabilities and experiences of maltreatment. TD: children with typical development. KW: Kruskal–Wallis test. D: Dunn–Bonferroni test. We have shown Bonferroni-corrected *p*-values.

### 3.3. Interoception Differences (Table 4)

A three-arm comparison showed significant differences in interoception (the heartbeat perception test scores) (*p* = 0.048), with the DM group scoring significantly lower than the TD group (*p* = 0.043). Significant differences were found in the MAIA self-control (*p* = 0.032), listening to the body (*p* = 0.014), and trust (*p* = 0.015) in the three-group comparison. The Bonferroni correction results indicated that group DD scored significantly lower than group TD in all three subsections (*p* = 0.027; *p* = 0.010; *p* = 0.013).

**Table 4:** Comparison of three groups’ MAIA scale scores (*n* = 24).

	Total (*n* = 24)	DM (*n* = 9)	DD (*n* = 8)	TD (*n* = 7)
Characteristics	Mean ± SD	Mean ± SD	Mean ± SD	Mean ± SD
MAIA				
Noticing	1.38 ± 1.15	1.17 ± 0.97	0.81 ± 1.01	2.29 ± 1.10
	KW = 5.844			
	*p* = 0.054			
Not-Distracting	3.11 ± 1.43	3.22 ± 1.51	3.25 ± 1.62	2.81 ± 1.26
	KW = 0.585			
	*p* = 0.746			
Not-Worrying	2.78 ± 0.98	2.93 ± 0.81	2.29 ± 1.09	3.14 ± 0.96
	KW = 1.708			
	*p* = 0.426			
Attention Regulation	1.79 ± 1.29	1.59 ± 1.22	1.27 ± 1.17	2.65 ± 1.22
	KW = 4.590			
	*p* = 0.101			
Emotional Awareness	2.87 ± 3.94	4.00 ± 6.20	1.45 ± 1.58	3.03 ± 0.86
	KW = 5.626			
	*p* = 0.06			
Self-Regulation	1.78 ± 1.40	1.72 ± 1.27	0.94 ± 0.81	2.83 ± 1.53
	KW = 6.904		D^DD-TD^ = 9.545	
	*p* = 0.032		*p*^DD-TD^ = 0.027	
Body Listening	1.26 ± 1.04	1.15 ± 0.82	0.58 ± 0.64	2.19 ± 1.09
	KW = 8.552		D^DD-TD^ = 10.536	
	*p* = 0.014		*p*^DD-TD^ = 0.010	
Trust	2.21 ± 1.53	1.93 ± 1.08	1.38 ± 1.69	3.52 ± 1.05
	KW = 8.366		D^DD-TD^ = 10.384	
	*p* = 0.015		*p*^DD-TD^ = 0.013	
Interoception	0.72 ± 0.16	0.56 ± 0.21	0.65 ± 0.18	0.81 ± 0.07
	KW = 6.074			D^TD-DM^ = 8.714
	*p* = 0.048			*p*^TD-DM^ = 0.043

DD: children with developmental disabilities. DM: foster home residents with developmental disabilities and experiences of maltreatment. TD: children with typical development. KW: Kruskal–Wallis test. D: Dunn–Bonferroni test. We have shown Bonferroni-corrected *p*-values.

### 3.4. Group Differences in Quizzes

No significant differences were found among the three groups in the mean quiz scores.

## 4. Discussion

This study is one of the few studies to use a VR classroom to identify the sensory characteristics of children with developmental disabilities and abusive experiences. Developmental disabilities and abuse experiences may overlap. Individuals can easily develop sensory idiosyncrasies and require early support. However, few studies have compared the sensory characteristics of children with developmental disabilities and abusive experiences to those of children with other conditions. We hope that this study will provide support that is tailored to children’s sensory characteristics.

### 4.1. Gaze

In the VR classroom environment, there was a significant difference between the three groups between 60 and 75 s, with the DM group spending significantly less time gazing at the “teacher” than the DD group, suggesting that the DM group’s interest in the “teacher” reduced after about 45 s and did not revert thereafter. The gazing pattern of the DM group in this study suggests that it is difficult to sustain interest in others. According to research regarding young adults exposed to harsh corporal punishment during childhood, the right anterior cingulate gyrus, which is involved in concentration, decision making, and empathy, showed a 16.9% reduction in volume [34]. It was thought that the DM group who experienced childhood abuse may have experienced some brain effects, leading to decreased concentration and interest in others. Additionally, the DD group consistently gazed at the “teacher” for a longer time from 0 to 75 s. Moreover, in a previous study, the DD group gazed at the “teacher” in the VR classroom significantly longer than the TD group, between 30 and 45 s [22]. It is conceivable that the DD group was interested in the “teacher” and consistently gazed at the object of interest. People with ADHD and ASD are known to be overly focused on their interests and have difficulty switching attention. This phenomenon is referred to as overconcentration or hyperfocus [35]. The diagnostic criteria for ADHD include an inattentive item listed as “often does not seem to listen when spoken to directly [28],” a behaviour that could be interpreted as evidence of hyperfocus [35]. Another effect of overconcentration in ASD children was that children with ASD were slower than TD children to disengage from an evolutionarily threatening snake image (disturbing visual stimulus), making it more difficult for them to detect the target flower image [36]. It is possible that the DD group in this study had ASD or ADHD and had difficulty diverting their attention from the visual stimulus of the teacher due to hyperfocus. In addition to this potential for hyperfocus, it has been noted that the pattern of attention is sticky in ASD children who are less interested in looking at emotional faces [37]. Our VR teacher’s facial expressions were always neutral, with no emotional changes, suggesting that the staring may have continued in the DD group, whereas the children in the other groups gradually lost interest. The DM group had a significantly longer gazing time than the DD and TD groups, possibly because the DM group lost interest in the “teacher” and shifted their interest to other visual stimuli and explored inside the VR classroom. It was thought that they were visually pursuing their new interests, that their attention was less likely to be sustained than that of the DD and TD groups, and that they may have been hypersensitive to the surrounding stimuli. Additionally, the very short gaze time directed at the “notebook” as well as the “teacher” suggests that they may have hardly noticed the audiovisual stimulus of the falling notebook. In the DM group in this study, they were likely unable to respond to other stimuli because they were absorbed in the further exploration of new stimuli. Nevertheless, their inability to allocate their cognition and senses dexterously to a variety of stimuli is thought to affect their academic performance. Among foster children in children’s homes in Japan, the subsection scores for social, attention, aggressive, externalising, and total problems on the Child Behaviour Checklist were significantly higher in the group that experienced abuse than in the group that did not experience abuse [38]. There were also significant differences in the items for poor academic performance [38]. Children with ASD and ADHD may also struggle academically without intellectual disabilities [34,35]. As children with abusive experiences and developmental disabilities often overlap, early support based on their sensory characteristics should be provided to prevent them from losing their academic confidence.

### 4.2. SSP

Concerning tactile sensitivity, the DD group scored significantly higher than the DM group. The DM group also scored lower than the TD group on tactile sensitivity, although the difference was not significant. In Atchinson et al. [16], where 900 children had experienced abuse, approximately 42% of the children had hypersensitivity to tactile sensitivity as assessed by the foster parents. Other studies hypothesised that for children who have experienced trauma from maltreatment, a lack of touch in childhood leads to a blunted sensitivity to touch [39]. In other words, tactile hypersensitivity or insensitivity may be induced in abused children during development to adapt to traumatic experiences, and further research is needed. Moreover, the total SSP score was significantly higher in the DM group than in the TD group, suggesting that sensory dysregulation cannot be ruled out.

The DD group in this study scored significantly higher than the TD group on taste/smell sensitivity, under-responsive/seeks sensation, and auditory filtering. A previous study of 3–6-year-old children with ASD found that compared to typically developed children, more than 50% differences of prevalence were reported on the under-responsive/seeks sensation, auditory filtering, and tactile sensitivity sections, as well as more than 40% differences in taste/smell sensitivity and visual/auditory sensitivity [40]. Therefore, the sensory characteristics exhibited by the DD group in this study were considered to be generally consistent with the results of previous studies. According to the Geneva Autism Cohort study, the factors most strongly associated with social impairment were under-responsive/seeks sensation and auditory filtering assessed using the SSP in a sample of children with ASD aged 3 to 6 [18]. It is likely that a small number of children in the DD group had social impairment and therefore might have obtained the lowest score of the performance test. Support tailored to their sensory characteristics is needed in the school setting.

### 4.3. Interoception

The DM group had the lowest interoceptive score in the heartbeat tracking task among the three groups, which was significantly different from that of the TD group. Trauma causes a sense of insecurity in one’s body and a disconnection from one’s sense of self [41]. The mind–body disconnection might be associated with the DM group’s low interoception. Garfinkel [21] noted that low interoceptive accuracy in individuals with ASD was linked to high anxiety levels. Additionally, more recent studies have found that it was interoceptive sensibility and not accuracy that was significantly associated with social anxiety in autistic young people [42]. The results of this study suggest that children who have maltreatment experience and/or developmental disabilities may have even lower interoceptive accuracy and interoceptive sensibility and therefore higher social anxiety. It is vital to pay attention to the sensory, emotional, and spiritual problems of children with trauma and disabilities and to provide them with the necessary care. For traumatised children, a supporting growth mindset could mitigate the negative impact of trauma on academic performance [43]. Therefore, it is necessary to support maltreated children’s mindset in school and community, in addition to previously recommended somatic sensory-based therapies and body–mind resetting methods [41].

Among the MAIA subscales, the means of “self-regulation”, “body listening”, and “trust” were significantly lower in the DD group than in the TD group; there were no significant differences in the DM group from the DD and TD groups. Regarding the children’s self-administered body perception, the DD group was considered to have the lowest. Whilst recent studies have paid attention to the role of negative performance beliefs and self-focused attention on social anxiety, only heightened interoceptive sensibility fully mediated the relationship between self-ratings of social performance and social anxiety in autistic young people [44]. Therefore, it is necessary to verify in the future whether relaxation methods, which are thought to be effective in reducing social anxiety [45], act on the body perception of developmentally disabled persons and help improve their academic performances.

### 4.4. Limitations of This Study and Future Issues

First, the number of participants was small, and the interpretation of the results obtained should be conducted with caution. For example, we did not identify an effect of diagnosis regarding the percentage of fixation on objects. Future studies should thus ensure a sufficient sample size and differentiate the ASD-only, ADHD-only, and co-occurrent groups to ensure the heterogeneity of neurodevelopmental disorders. However, this is a study that used the pioneering method of VR classrooms to assess sensory characteristics of children who experienced abuse and developmental disabilities, and we believe that the findings are valuable. Second, because we did not obtain information on the subtype of abuse or the age at which the abuse occurred, we were unable to account for differences in participants’ sensory characteristics due to these influences. Third, there was a difference in the assessors in the SSP; for the DM group, they were institutional staff and may have had less time with the children than the parents, which may have influenced the results. Despite these limitations, we believe that the results of this study provided us with findings that can be used for future mental health research and child education.

## 5. Conclusions

The results of this study indicate that the children in the DM and DD groups may have different kinds of gazes at objects and body sensations in VR classrooms. Furthermore, the results of this study suggest that children may have difficulty understanding instructions in a school environment where audiovisual stimuli are nearby. The present results also have implications for future research, as the DM group had difficulty maintaining attention to the VR teacher, whereas the DD group spent considerably more time looking at the same object, suggesting that future research on how to focus and gaze according to the neurodevelopmental diagnosis and traumatised experiences would be useful. Moreover, the lower level of awareness of tactile sensations and interoception among the DM group suggests that an evaluation of the interoceptive awareness and sensations of children is needed in applying tailored care to restore the body–mind connection. The usefulness of VR classrooms as a method for approaching these sensory and cognitive atypicalities has been suggested.

## Figures and Tables

**Table 1:** Comparison of three groups’ sociodemographic characteristics (*n* = 24).

	Total (*n* = 24)	DM (*n* = 9)	DD (*n* = 8)	TD (*n* = 7)
Characteristics	Mean ± SD	Mean ± SD	Mean ± SD	Mean ± SD
Age	13.08 ± 1.84	13.22 ± 2.59	12.50 ± 1.60	13.57 ± 0.54
	KW = 2.144			
	*p* = 0.342			
Gender				
Male	14 (58.3%)	3 (33.3%)	6 (75.0%)	5 (71.4%)
Female	10 (41.7%)	6 (66.7%)	2 (25.0%)	2 (28.6%)
	* *p* > 0.05			

DD: children with developmental disabilities. DM: foster home residents with developmental disabilities and experiences of maltreatment. TD: children with typical development. KW: Kruskal–Wallis test. * In the Bonferroni-corrected z-test, there was no significant difference in sex between DD, TD, and DM.

## Data Availability

The data presented in this study are available on request from the corresponding author. The data are not publicly available due to restrictions on ethical considerations.
